# Percutaneous Transhepatic Cholangiodrainage (PTCD)-Related Hepatitis B Virus Reactivation in Obstructive Jaundice

**DOI:** 10.3390/jcm15093263

**Published:** 2026-04-24

**Authors:** Chao Chen, Zijian Liu, Yanqiao Ren, Tianyou Shao, Jinghong Yao

**Affiliations:** 1 Department of Radiology, Union Hospital, Tongji Medical College, Huazhong University of Science and Technology, Wuhan 430022, China; 15082090430@163.com (C.C.); 2022xh0059@hust.edu.cn (Y.R.); 2 Department of Anatomy, School of Basic Medical Sciences, Tongji Medical College, Huazhong University of Science and Technology, Wuhan 430030, China; liuzj@mail.hust.edu.cn; 3 Department of Interventional Vascular, Jiang Xi Province Chest Hospital, Nanchang 330006, China; 18379425656@163.com; 4 Department of Infectious Diseases, Union Hospital, Tongji Medical College, Huazhong University of Science and Technology, Wuhan 430022, China

**Keywords:** percutaneous transhepatic cholangiodrainage, obstructive jaundice, HBV reactivation, antiviral therapy

## Abstract

**Background:** Obstructive jaundice is a common clinical condition, often caused by malignant tumors such as hepatocellular carcinoma (HCC) or cholangiocarcinoma (CCA). Percutaneous transhepatic cholangiodrainage (PTCD) is a widely used intervention method to relieve biliary obstruction in patients with obstructive jaundice; however, the impact of PTCD on hepatitis B virus (HBV) reactivation has not been thoroughly studied. **Methods:** A retrospective analysis was conducted from January 2016 to December 2024 on 235 patients with obstructive jaundice who underwent PTCD. Demographic, clinical, and procedural data were collected, and multivariate logistic regression was used to identify risk factors for HBV reactivation. Additionally, Cox regression was used to evaluate the time-to-reactivation variables. **Results:** The HBV reactivation rate in the PTCD group was 21.7%, significantly higher than the 8.9% in the non-PTCD group. Key risk factors for HBV reactivation in the PTCD group included the absence of antiviral prophylaxis, postoperative infection, elevated preoperative HBV-DNA levels, and multiple biliary punctures. Moreover, Cox regression revealed that a lack of antiviral therapy and postoperative infection were associated with earlier HBV reactivation. **Conclusions:** PTCD significantly increases the risk of HBV reactivation in patients with obstructive jaundice, especially in those with high preoperative HBV-DNA levels and without antiviral prophylaxis. Early detection of HBV reactivation and the initiation of antiviral therapy are critical to improving patient outcomes. These findings underscore the need for careful monitoring of HBV status in patients undergoing PTCD.

## 1. Introduction

Obstructive jaundice is a prevalent clinical condition with a generally poor prognosis, primarily resulting from malignant neoplasms such as hepatocellular carcinoma (HCC), cholangiocarcinoma (CCA), or pancreatic cancer. These tumors exert pressure on the intrahepatic or extrahepatic bile ducts, leading to biliary stasis. The typical clinical presentation of obstructive jaundice includes scleral and cutaneous jaundice, pruritus, and a range of other symptoms that severely impact the quality of life of affected patients, particularly those with cancer [1,2]. Traditionally, the management of obstructive jaundice has relied on surgical resection or pharmacological interventions aimed at relieving biliary pressure [3]. However, early detection of these malignancies remains a significant clinical challenge, resulting in a substantial proportion of patients being ineligible for surgical intervention. Furthermore, prolonged pharmacological treatment often exacerbates the metabolic load on the liver and kidneys, complicating patient management and outcomes [4,5,6].

Percutaneous transhepatic cholangiodrainage (PTCD) has emerged as a minimally invasive intervention, typically performed under ultrasound or digital subtraction angiography (DSA) guidance. This procedure involves percutaneous biliary puncture, ductal dilation, and external drainage to alleviate biliary obstruction. The primary etiology of obstructive jaundice is malignant compression caused by HCC or CCA, with a substantial number of patients with obstructive jaundice having a concurrent or prior history of hepatitis B virus (HBV) infection. While PTCD offers several advantages as a minimally invasive approach with broad clinical applicability, it is not without risks, with postoperative complication rates remaining substantial [7]. Importantly, this invasive procedure may induce immune system alterations, including immune stress or transient immunosuppression, which can, in turn, potentially trigger HBV reactivation [8,9]. Moreover, the standard treatment protocols for advanced-stage malignancies—often combining systemic chemotherapy, transarterial chemoembolization (TACE), and immunotherapy—are known to elevate the risk of HBV reactivation. While both cytotoxic chemotherapy and immunosuppressive therapies have been shown to induce HBV reactivation, the specific risk posed by PTCD has yet to be quantified [10,11,12,13,14]. Notably, prior studies have indicated that HBV reactivation can lead to a spectrum of hepatic injury, from mild biochemical abnormalities to fulminant hepatic failure in severe cases [15]. While the risk of HBV reactivation associated with chemotherapy and immunotherapy has been well established, there is limited evidence regarding the potential for HBV reactivation following PTCD, a localized intervention. No studies have explored or quantified the risk factors associated with PTCD or the time-course of HBV reactivation following this procedure.

Therefore, the aim of this study is to assess the impact of PTCD on the reactivation of hepatitis B virus in patients with hepatocellular carcinoma or cholangiocarcinoma following TACE. Additionally, the aim is to quantify the incidence of HBV reactivation post PTCD and to identify the risk factors associated with this phenomenon. The findings of this research will provide important insights that may inform the clinical management of obstructive jaundice, particularly in patients requiring biliary drainage interventions, and could contribute to the optimization of treatment strategies for this patient population.

## 2. Methods

### 2.1. Patients

We conducted a retrospective analysis of 235 consecutive patients diagnosed with obstructive jaundice who underwent PTCD at our institution between January 2016 and December 2024. The inclusion criteria for the study were as follows: (1) patients aged ≥18 years; (2) serological evidence of prior hepatitis B virus (HBV) infection, confirmed by the presence of HBsAg and/or anti-HBc antibodies; (3) the availability of complete clinical and follow-up data; and (4) primary malignancy identified as HCC or CCA. Exclusion criteria included the following: (1) the presence of severe concurrent cardiopulmonary, hepatic, or renal insufficiency, defined as Child–Pugh class C or its equivalent (severe concurrent cardiopulmonary—New York Heart Association (NYHA) functional class III or IV, or requiring continuous oxygen therapy; severe renal insufficiency—estimated glomerular filtration rate (eGFR) < 30 mL/min/1.73 m^2^ or requiring renal replacement therapy); (2) non-malignant causes of obstructive jaundice, such as alcoholic hepatitis, primary biliary cholangitis, or autoimmune hepatitis; and (3) the use of immunosuppressive therapy. Patients who had previously received immune regulation or conventional chemotherapy were not automatically excluded; however, at least 6 months were required to have elapsed since the completion of such therapies before enrollment, in order to minimize the potential impact of prior treatments on HBV reactivation. Additionally, we collected comparable clinical data from an equal number of patients with HCC or CCA who did not undergo PTCD during the same study period. These patients were selected using the same inclusion and exclusion criteria as the PTCD group to ensure a consistent and representative comparison.

**Figure 1:**
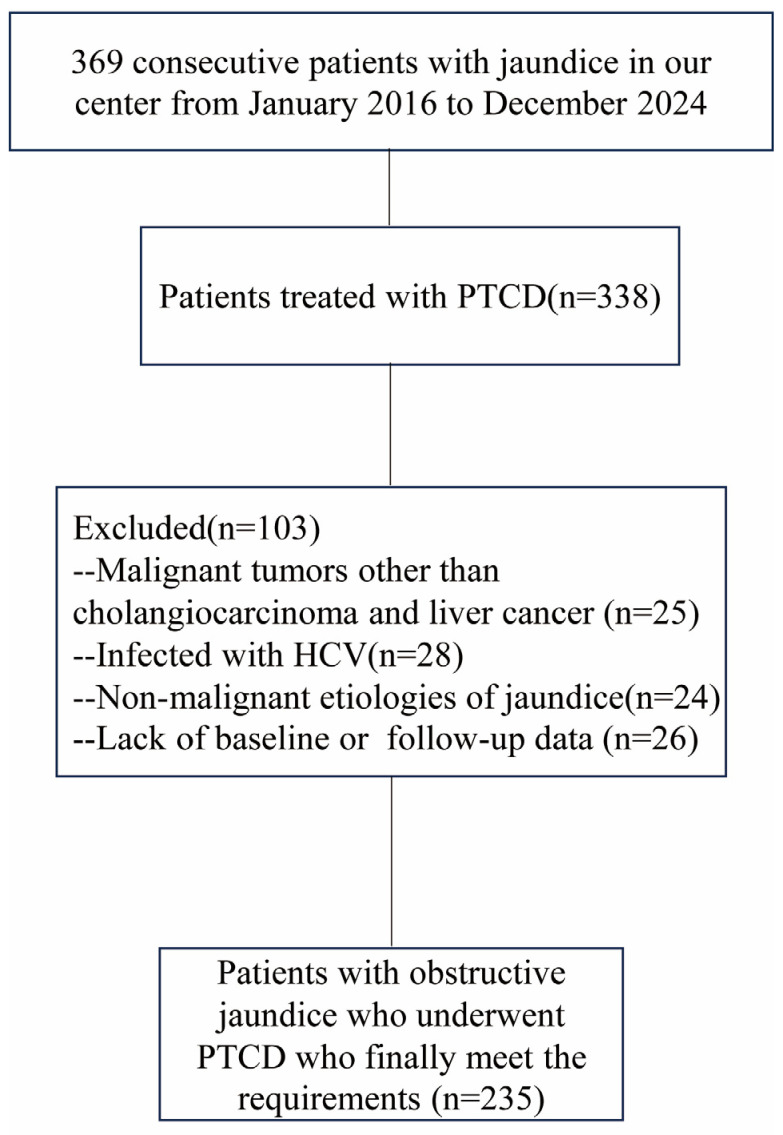
A flowchart depicting the selection of patients who received PTCD. **Note.** PTCD: Percutaneous transhepatic cholangiodrainagel; HCV: Hepatitis C Virus.

HBV reactivation was defined using the following criteria: (1) for HBsAg-positive patients, a ≥2 log_10_ IU/mL increase in HBV DNA levels compared to baseline; (2) for HBsAg-negative/anti-HBc-positive patients, HBV DNA levels ≥ 100 IU/mL or the appearance of HBsAg [16]. This study adhered to the principles outlined in the Declaration of Helsinki, and informed consent was waived due to the retrospective nature of the study. A flowchart outlining the patient selection process is provided in Figure 1. Additionally, we collected comparable clinical data from an equal number of patients with liver and bile duct cancers who did not undergo PTCD within the same time period. These patients were selected using the same inclusion and exclusion criteria applied to the PTCD group, ensuring a consistent and representative comparison. Concurrently, for patients subjected to PTCD, data regarding the procedure duration, number of puncture attempts, and the occurrence of postoperative complications such as infection, hemorrhage, or bile leakage were collected.

### 2.2. Baseline Clinical Data

The baseline characteristics of the study participants included demographic information (age, sex), behavioral factors (such as smoking and alcohol consumption history), the etiology of biliary obstruction, and pre-procedural antiviral medication usage. Laboratory parameters were also recorded, consisting of the following: (1) pre- and postoperative (4-week) liver function tests, which included alanine aminotransferase (ALT), aspartate aminotransferase (AST), total bilirubin, prothrombin time, and albumin levels, and (2) serial HBV-DNA viral load measurements, obtained preoperatively and during each scheduled follow-up visit. Antiviral prophylaxis, primarily including entecavir or tenofovir, was administered to patients based on clinical guidelines.

### 2.3. PTCD Procedure

The PTCD procedure was conducted by experienced surgeons (≥5 years of practice). Initially, the appropriate bile ducts were identified using either ultrasound or digital subtraction angiography (DSA). An 18-gauge (200 mm) puncture needle was used to access the targeted bile duct, with cholangiography performed through the needle by injecting a contrast medium to clearly define the site of biliary obstruction. Under image guidance, an 8-French pigtail drainage catheter was advanced into the obstructed bile duct. The correct placement of the external drainage tube was confirmed by administering a residual contrast agent through the catheter. Finally, the drainage tube was securely affixed to ensure its stability and proper functioning throughout the drainage period.

### 2.4. Follow-Up and Assessment

Patients who underwent PTCD were followed up for retrospective data collection until 30 May 2025. The primary focus of the follow-up data collection was on HBV DNA levels, which were monitored closely. Additionally, the follow-up aimed to track any reactivation of HBV, as well as the specific timing of such events. The primary endpoint of the study was HBV reactivation, and the time to HBV reactivation was calculated from the initiation of treatment until the detection of reactivation. HBV-DNA levels were originally measured in copies/mL, and the threshold for “high load” was defined as >1 × 10^4^ copies/mL. Follow-up for HBV reactivation was conducted at 4-week intervals after PTCD, continuing until reactivation occurred or for a maximum duration of 24 weeks.

### 2.5. Statistical Analyses

Continuous variables are presented as mean ± standard deviation or median with interquartile range (IQR), as appropriate based on data distribution. In preliminary univariate analyses, variables showing marginal significance (*p* < 0.10) in *t*-tests (for continuous variables) or chi-square tests (for categorical variables) were selected for inclusion in subsequent multivariate logistic regression modeling. Prognostic factors for HBV reactivation time were assessed using univariate and multivariate cox regression analyses. All statistical analyses were conducted using SPSS software (version 29.0; IBM Corp., Armonk, NY, USA), with supplementary analyses and graphical representations generated using GraphPad Prism 10.0 (GraphPad Software, San Diego, CA, USA). A two-sided *p*-value < 0.05 was considered statistically significant for all final analyses.

## 3. Results

### 3.1. Patient Characteristics

**After applying the inclusion and exclusion criteria, a total of 470 patients were enrolled in this study, with 235 patients in each group.** No significant differences in clinical baseline characteristics were observed between the two groups, except for total bilirubin levels. The PTCD cohort had a mean age of 58.23 ± 12.18 years, with a male predominance of 78.7% (n = 185). A majority of patients reported a history of alcohol consumption (66.9%, n = 157) and smoking (65.1%, n = 153). The primary tumor types were predominantly CCA at 65.1% (n = 153), followed by HCC at 34.9% (n = 82). 42.1% (99/235) in the PTCD group and 39.6% (93/235) in the non-PTCD group were classified as Child–Pugh class B. Among the 235 patients in the PTCD group, 198 (84.3%) were HBsAg-positive and 37 (15.7%) were HBsAg-negative/anti-HBc-positive. Baseline HBV DNA was undetectable in 65 patients (27.7%) in the PTCD group and 58 patients (24.7%) in the non-PTCD group. Preoperative assessment revealed that 34.0% (n = 80) of patients had elevated HBV-DNA levels, and 51.5% (n = 121) were receiving antiviral therapy. All PTCD procedures were technically successful, with a mean operative duration of 14.5 ± 3.27 min. The success rate of first-attempt biliary puncture was 90.6% (n = 213). Procedure-related complications were observed in a minority of patients: biliary hemorrhage in 6.4% (n = 15), infection in 15.7% (n = 37), and bile leakage in 2.1% (n = 5). All complications were managed conservatively and resolved without further interventions. HBV reactivation occurred in 21.7% (51/235) of patients undergoing PTCD, compared to 8.9% (21/235) of patients who did not undergo PTCD. The clinical baseline characteristics of both groups are summarized in Table 1, and detailed information regarding the intraoperative procedures and treatment specifics for the PTCD group can be found in Table 2.

### 3.2. Clinical Factors Related to HBV Reactivation

In the overall patient cohort, univariate logistic regression analysis identified several factors associated with HBV reactivation. These included hyperbilirubinemia (OR = 1.004, 95% CI [1.000–1.008], *p* = 0.075), younger age (OR = 0.564, 95% CI [0.333–0.957], *p* = 0.034), and undergoing percutaneous transhepatic cholangiodrainage (PTCD) (OR = 2.825, 95% CI [1.638–4.781], *p* < 0.001). Variables that reached statistical significance or near-significance in the univariate analysis were subsequently included in a multivariate logistic regression model. The multivariate analysis demonstrated that younger age (OR = 0.582, 95% CI [0.341–0.994], *p* = 0.048) and undergoing PTCD (OR = 2.856, 95% CI [1.543–5.287], *p* < 0.001) were independently associated with an increased risk of HBV reactivation (Table 3). Focusing specifically on patients who underwent PTCD, univariate logistic regression identified several procedural and clinical factors significantly associated with HBV reactivation. These included the absence of antiviral prophylaxis (OR = 0.344, 95% CI [0.178–0.665], *p* = 0.002), longer PTCD operation time (OR = 2.076, 95% CI [1.105–3.900], *p* = 0.023), postoperative infection (OR = 4.737, 95% CI [2.248–9.980], *p* < 0.001), elevated preoperative HBV-DNA levels (HR = 2.032, 95% CI [1.079–3.827], *p* = 0.028), hemobilia (HR = 3.500, 95% CI [1.204–10.171], *p* = 0.021), and multiple bile duct punctures (OR = 7.526, 95% CI [2.917–19.421], *p* < 0.001). These variables were subsequently included in a multivariate logistic regression model to identify independent predictors of HBV reactivation in the PTCD cohort. The results revealed that a lack of preoperative antiviral prophylaxis (OR = 0.119, 95% CI [0.040–0.349], *p* < 0.001), postoperative infection (OR = 3.230, 95% CI [1.379–7.564], *p* = 0.007), high baseline HBV-DNA load (OR = 6.271, 95% CI [2.138–18.395], *p* < 0.001), and multiple biliary tract punctures (OR = 4.206, 95% CI [1.323–13.375], *p* = 0.015) were independently associated with an increased likelihood of HBV reactivation. Detailed results are presented in Table 4. The relationship between preoperative HBV viral load, antiviral prophylaxis, and subsequent HBV reactivation was further analyzed. Among patients with low baseline HBV-DNA levels, preoperative antiviral therapy significantly reduced the incidence of reactivation compared with untreated patients (5.6% vs. 23.8%, *p* = 0.04). In patients with high baseline viral loads, preoperative antiviral treatment was even more protective, reducing reactivation rates from 84.6% in untreated patients to 19.4% in treated patients (*p* < 0.001). These findings underscore the critical protective role of antiviral prophylaxis in preventing HBV reactivation across all strata of baseline viral loads.

### 3.3. Prognostic Factors for HBV Reactivation Time

**Figure 2:**
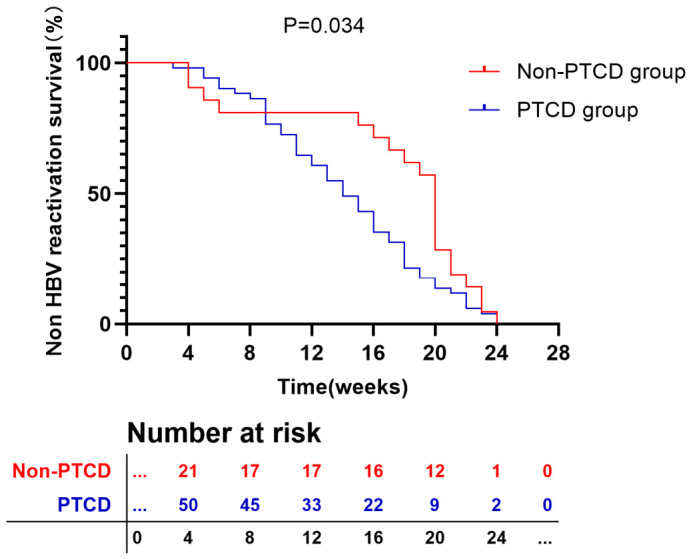
Kaplan–Meier Curve of HBV reactivation time.

In patients undergoing PTCD who experienced HBV reactivation, the median time to reactivation was 14 weeks, with a range of 3 to 24 weeks. And the median time for HBV reactivation in the non PTCD group is 20 weeks, with a range of 4 to 24 weeks. There was a statistically significant difference in HBV reactivation time between the two groups (*p* = 0.034) (Figure 2). In PTCD group, univariate Cox regression analysis led to the identification of several factors significantly associated with the timing of HBV reactivation. These included preoperative antiviral therapy (HR = 0.299, 95% CI [0.158–0.566], *p* < 0.001), a longer PTCD operation time (HR = 2.480, 95% CI [1.357–4.533], *p* = 0.003), postoperative infection (HR = 3.615, 95% CI [1.865–7.005], *p* < 0.001), and hemobilia (HR = 2.201, 95% CI [0.973–4.979], *p* = 0.058). Multivariate Cox regression analysis further demonstrated that the absence of preoperative antiviral therapy (HR = 0.371, 95% CI [0.186–0.742], *p* = 0.005) and the occurrence of postoperative infection (HR = 2.317, 95% CI [1.099–4.884], *p* = 0.027) were independently associated with earlier HBV reactivation. These findings suggest that both preoperative antiviral prophylaxis and the prevention of postoperative infection are critical for delaying or mitigating HBV reactivation following PTCD. Detailed results are presented in Table 5.

## 4. Discussion

PTCD has become a widely adopted, minimally invasive intervention for managing malignant obstructive jaundice, particularly in patients with CCA or HCC. By effectively reducing biliary tract pressure and lowering serum bilirubin levels, PTCD improves patients’ tolerance to subsequent anticancer therapies. However, its role in promoting HBV reactivation remains underexplored. The aim of this retrospective study was to investigate the association between PTCD and HBV reactivation, focusing primarily on a cohort of patients who underwent PTCD for obstructive jaundice secondary to CCA or HCC.

We included 235 patients who underwent PTCD and 235 who did not, with 51 patients (21.7%) in the PTCD group experiencing HBV reactivation. This rate was significantly higher than the approximately 10% observed in previous studies [11,17,18] and the 8.9% observed in the non-PTCD group of this study. Given this notable discrepancy, we hypothesized that PTCD might facilitate HBV reactivation. Univariate logistic regression analysis confirmed that PTCD and high preoperative bilirubin levels were associated with an increased risk of HBV reactivation. Further multivariate analysis identified several significant risk factors for HBV reactivation, including the absence of preoperative antiviral therapy, postoperative infection, high preoperative HBV DNA load, and multiple biliary punctures. These findings suggest that the invasive nature of PTCD, particularly multiple biliary punctures, may induce transient immune stress or immunosuppression. Repeated biliary manipulation during PTCD could disrupt the liver’s immune-privileged status, promoting the release of cytokines, such as interleukin-6 (IL-6), which may facilitate viral replication [19,20]. Previous studies have established immunosuppression as a key factor in HBV reactivation [21,22,23], and this effect may be amplified in patients with elevated baseline HBV DNA levels or those not receiving antiviral prophylaxis. Our analysis further demonstrated that antiviral therapy significantly reduced the rate of HBV reactivation, irrespective of the patient’s preoperative HBV DNA load. Notably, in patients with high HBV DNA levels, reactivation rates exceeded 80% in the absence of antiviral prophylaxis, underscoring the critical role of antiviral therapy in these patients. These findings highlight the importance of initiating or continuing antiviral therapy in patients undergoing PTCD who have high HBV DNA levels, regardless of whether the HBV infection is newly acquired or pre-existing. In this cohort, nearly half of the patients (48.5%) did not receive antiviral prophylaxis before PTCD, and 34.0% had baseline HBV-DNA levels above 1 × 10^4^ copies/mL—a threshold widely considered to indicate a need for antiviral therapy. Several factors may explain this less than optimal pretreatment status. First, the study included patients enrolled as early as 2016, when institutional practices regarding antiviral prophylaxis for invasive procedures were less standardized and adherence to HBV management guidelines varied among clinicians. Second, some patients with high viral loads were referred for PTCD on an urgent basis, leaving insufficient time to initiate antiviral therapy beforehand. Third, a subset of patients had been lost to follow-up for chronic HBV management prior to their cancer diagnosis, resulting in missed opportunities for timely antiviral intervention. Notably, after 2020, our center adopted a more stringent protocol recommending universal antiviral prophylaxis for all HBsAg-positive patients undergoing PTCD, which led to a marked improvement in adherence.

It is worth noting that, although this study showed that conventional antiviral therapy (such as nucleotide analogues) significantly reduced the risk of reactivation in patients with preoperative low-load HBV-DNA, certain novel therapies targeting specific immune pathways or viral replication pathways (such as certain immunomodulators or experimental antiviral strategies) may induce HBV reactivation by altering immune balance [24,25]. We also explored the temporal characteristics of HBV reactivation following PTCD. Our analysis revealed that postoperative infection and the absence of preoperative antiviral therapy were significant predictors of earlier HBV reactivation. Interestingly, although high baseline HBV DNA levels and multiple biliary punctures were identified as risk factors for reactivation, they did not influence the timing of reactivation. This temporal dissociation could be explained by the fact that most patients with high viral loads received antiviral prophylaxis, which, while not completely preventing reactivation, appeared to modulate its timing. The limited number of biliary punctures in this cohort (mostly 1–2) may have caused insufficient trauma to accelerate reactivation significantly.

Regarding PTCD-related complications, while bile leakage is a serious procedural adverse event, it showed no significant association with HBV reactivation in this study. This negative result may be attributed to the low incidence of bile leakage in our cohort, which limited statistical power. Additionally, the widespread use of prophylactic antiviral therapy likely mitigated any potential reactivation risk associated with bile leakage.

The median time to HBV reactivation in this study was 14 weeks (range: 3–24 weeks), which was notably earlier than that reported in prior studies and non-PTCD group [18,26,27]. This temporal pattern suggests that PTCD may pose a higher risk for HBV reactivation compared to other clinical contexts. Given the high prevalence of HBV infection in our study population, we recommend routine HBV-DNA testing before PTCD and during follow-up, even for patients who are hepatitis B surface antigen (HBsAg)-negative. For those with detectable HBV DNA, prompt initiation of antiviral therapy is crucial to reduce the risk of reactivation and its associated complications.

Several limitations of this study should be acknowledged. First, as a single-center, retrospective analysis, our results may be subject to selection bias, particularly due to potential loss to follow-up. Second, the predominance of patients with cancer in the cohort might introduce bias, as disease progression and mortality may have precluded HBV reactivation in some individuals, potentially affecting our estimates. Third, although we identified clinical factors associated with HBV reactivation, the underlying biological mechanisms remain unclear and warrant further investigation. Fourth, we were unable to include a comparator group of patients who underwent endoscopic retrograde cholangiography (ERC) for malignant biliary obstruction, as ERC procedures are performed in a different department and data integration was not feasible within the scope of this retrospective study. Future multicenter studies that include ERC cohorts would help clarify whether the observed reactivation risk is specific to PTCD or generalizable to other biliary interventions. Fifth, the number of HBV-positive patients who underwent PTCD for benign obstructive jaundice (e.g., biliary stones, strictures) during the study period was too small (n = 12) to allow meaningful statistical comparison; thus, our findings primarily apply to patients with malignant etiologies. Finally, the widespread use of antiviral prophylaxis in our study population may have obscured the true impact of certain PTCD-related procedural factors on HBV reactivation risk. However, the multi-center collaboration and rigorous sensitivity analyses conducted as part of this study enhance the validity of our findings.

In conclusion, our study demonstrates that PTCD is significantly associated with HBV reactivation, particularly in patients with high preoperative HBV DNA loads who do not receive antiviral prophylaxis. These findings emphasize the critical importance of implementing standardized HBV-DNA monitoring before and after PTCD. Such monitoring facilitates timely detection and management of HBV reactivation, thereby minimizing potentially adverse clinical outcomes associated with viral reactivation.

## Figures and Tables

**Table 1:** Clinical features.

Characteristics	PTCD (n = 235)	Non-PTCD (n = 235)	*p* Value
Gender			0.245
Male	184 (78.3%)	191 (82.6%)	
Female	51 (21.7%)	41 (17.4%)	
Age (years)	58.23 ± 12.18	58.58 ± 11.64	0.751
Smoking history			0.394
Yes	149 (63.4%)	140 (59.6%)	
No	86 (36.6%)	95 (40.4%)	
Drinking history			0.248
Yes	157 (66.8%)	145 (61.7%)	
No	78 (33.2%)	90 (38.3%)	
HBV-DNA			0.638
Undetectable	65 (27.7%)	58 (24.7%)	
Detectable ≤ 1 × 10^4^	90 (38.3%)	88 (37.4%)	
>1 × 10^4^	80 (34.0%)	89 (37.9%)	
Etiology			0.444
CCA	153 (65.1%)	145 (61.7%)	
HCC	82 (34.9%)	90 (38.3%)	
Child-Pugh			0.573
A	93 (39.6%)	99 (42.1%)	
B	142 (60.4%)	136 (57.9%)	
HBV infection			0.102
HBsAg and anti-HBc (+)	210 (89.4%)	198 (84.3%)	
Anti-HBc (+) only	25 (10.6%)	37 (15.7%)	
HBV reactivation			<0.001
Yes	51 (21.7%)	21 (8.9%)	
No	184 (78.3%)	214 (91.1%)	
ALT (IU/L)	56.47 ± 58.47	54.56 ± 35.59	0.670
AST (IU/L)	57.35 ± 37.32	51.96 ± 36.19	0.112
TB (μmol/L)	71.90 ± 61.10	16.07 ± 7.12	<0.001
Alb (IU/L)	36.55 ± 4.89	36.70 ± 5.27	0.753
PT (s)	14.16 ± 1.50	14.08 ± 1.26	0.510

TB: Total bilirubin; PT: Prothrombin time; AST: Aspartate aminotransferase; ALT: Alanine aminotransferase; Alb: Albumin; CCA: Cholangiocarcinoma; HCC: Hepatocellular carcinoma; HBV: Hepatitis B Virus.

**Table 2:** Treatment information for PTCD patients.

Characteristics	No.	Percentage
**Bile duct punctures**		
1	213	90.6
≥2	22	9.4
**Biliary leaks**		
Yes	5	2.1
No	230	97.5
**Hemobilia**		
Yes	15	6.4
No	220	93.6
**Infection**		
Yes	37	15.7
No	198	84.3
**Antiviral therapy**		
Yes	121	51.5
No	114	48.5

**Table 3:** Factors related to HBV reactivation.

Variable	Univariate Analysis			Multivariate Analysis		
	OR	95%CI	*p*	OR	95%CI	*p*
**Gender** Male/Female	0.912	0.490–1.696	0.770			
**Age (years)** ≤60/>60	0.564	0.333–0.957	0.034	0.582	0.341–0.994	0.048
**Smoking history** Yes/No	1.019	0.609–1.706	0.943			
**Drinking history** Yes/No	1.173	0.700–1.966	0.545			
**ALT**	0.996	0.989–1.003	0.237			
**AST**	1.000	0.993–1.007	0.976			
**Alb**	1.005	0.954–1.057	0.862			
**PT**	0.888	0.731–1.079	0.231			
**TB**	1.004	1.000–1.008	0.075	1.000	0.994–1.005	0.852
**HBV-DNA** high/low load	1.160	0.692–1.944	0.573			
**PTCD** Yes/No	2.825	1.638–4.871	<0.001	2.856	1.543–5.287	<0.001
**Etiology** CCA/HCC	0.908	0.537–1.537	0.720			

ALT: Alanine aminotransferase; AST: Aspartate aminotransferase; PT: Prothrombin time; TB: Total bilirubin; Alb: Albumin; CCA: Cholangiocarcinoma; HCC: Hepatocellular carcinoma; PTCD: Percutaneous transhepatic cholangiodrainagel.

**Table 4:** Factors related to HBV reactivation in the PTCD group.

Variable	Univariate Analysis			Multivariate Analysis		
	OR	95%CI	*p*	OR	95%CI	*p*
**Gender** Male/Female	1.010	0.475–2.146	0.979			
**Age (years)** <60/≥60	1.322	0.710–2.462	0.378			
**Smoking history** Yes/No	1.153	0.610–2.183	0.661			
**Drinking history** Yes/No	0.900	0.462–1.751	0.755			
**ALT**	0.996	0.989–1.003	0.296			
**AST**	0.999	0.991–1.008	0.831			
**Alb**	1.021	0.958–1.089	0.516			
**PT**	0.888	0.704–1.119	0.313			
**TB**	1.003	0.998–1.008	0.221			
**Antiviral therapy** Yes/No	0.344	0.178–0.665	0.002	0.119	0.040–0.349	<0.001
**Operation time** **>14/≤14**	2.076	1.105–3.900	0.023	1.161	0.548–2.457	0.697
**Infection** Yes/No	4.737	2.248–9.980	<0.001	3.230	1.379–7.564	0.007
**HBV-DNA** High/low load	2.032	1.079–3.827	0.028	6.271	2.138–18.395	<0.001
**Bile duct punctures** ≥2/1	7.526	2.917–19.421	<0.001	4.206	1.323–13.375	0.015
**Hemobilia** Yes/No	3.500	1.204–10.171	0.021	1.286	0.320–5.164	0.723
**Biliary leaks** Yes/No	2.463	0.400–15.150	0.331			
**Etiology** CCA/HCC	1.411	0.747–2.668	0.289			

ALT: Alanine aminotransferase; AST: Aspartate aminotransferase; PT: Prothrombin time; TB: Total bilirubin; Alb: Albumin; CCA: Cholangiocarcinoma; HCC: Hepatocellular carcinoma.

**Table 5:** Univariate and multivariate analysis of factors associated with HBV reactivation time in the PTCD group.

Variable	Univariate Analysis			Multivariate Analysis		
	HR	95%CI	*p*	HR	95%CI	*p*
**Gender** Male/female	0.880	0.447–1.730	0.711			
**Age (years)** <60/≥60	1.417	0.795–2.524	0.237			
**Smoking history** Yes/No	0.948	0.538–1.669	0.852			
**Drinking history** Yes/No	1.301	0.704–2.403	0.401			
**ALT**	1.003	0.996–1.010	0.368			
**AST**	1.001	0.994–1.009	0.727			
**Alb**	1.009	0.946–1.077	0.789			
**PT**	0.984	0.774–1.252	0.897			
**TB**	1.003	0.999–1.008	0.164			
**Antiviral therapy** Yes/No	0.299	0.158–0.566	<0.001	0.371	0.186–0.742	0.005
**Operation time (min)** **>14/≤14**	2.480	1.357–4.533	0.003	1.824	0.940–3.539	0.076
**Infection** Yes/No	3.615	1.865–7.005	<0.001	2.317	1.099–4.884	0.027
**HBV-DNA** High/low load	0.935	0.535–1.632	0.813			
**Bile duct punctures** ≥2/1	1.696	0.894–3.216	0.106			
**Hemobilia** Yes/No	2.201	0.973–4.979	0.058	1.220	0.504–2.956	0.659
**Biliary leaks** Yes/No	0.445	0.100–1.974	0.287			
**Etiology** CCA/HCC	0.737	0.446–1.219	0.234			

## Data Availability

The anonymized data supporting the findings of this study are available from the corresponding author upon reasonable request.
